# Cholesterol lowering effects of mono-lactose-appended β-cyclodextrin in Niemann–Pick type C disease-like HepG2 cells

**DOI:** 10.3762/bjoc.11.224

**Published:** 2015-11-03

**Authors:** Keiichi Motoyama, Yumi Hirai, Rena Nishiyama, Yuki Maeda, Taishi Higashi, Yoichi Ishitsuka, Yuki Kondo, Tetsumi Irie, Takumi Era, Hidetoshi Arima

**Affiliations:** 1 Graduate School of Pharmaceutical Sciences, Kumamoto University, 5-1 Oe-honmachi, Chuo-ku, Kumamoto 862-0973, Japan; 2 Program for Leading Graduate Schools “HIGO (Health life science: Interdisciplinary and Glocal Oriented) Program”, Kumamoto University, 5-1 Oe-honmachi, Chuo-ku, Kumamoto 862-0973, Japan; 3 Department of Cell Modulation, Institute of Molecular Embryology and Genetics, Kumamoto University, 2-2-1 Honjo, Chuo-ku, Kumamoto 860-0811, Japan

**Keywords:** asialoglycoprotein receptor, cholesterol, cyclodextrin, lactose, Niemann–Pick disease type C

## Abstract

The Niemann–Pick type C disease (NPC) is one of inherited lysosomal storage disorders, emerges the accumulation of unesterified cholesterol in endolysosomes. Currently, 2-hydroxypropyl-β-cyclodextrin (HP-β-CyD) has been applied for the treatment of NPC. HP-β-CyD improved hepatosplenomegaly in NPC patients, however, a high dose of HP-β-CyD was necessary. Therefore, the decrease in dose by actively targeted-β-CyD to hepatocytes is expected. In the present study, to deliver β-CyD selectively to hepatocytes, we newly fabricated mono-lactose-appended β-CyD (Lac-β-CyD) and evaluated its cholesterol lowering effects in NPC-like HepG2 cells, cholesterol accumulated HepG2 cells induced by treatment with U18666A. Lac-β-CyD (degree of substitution of lactose (DSL) 1) significantly decreased the intracellular cholesterol content in a concentration-dependent manner. TRITC-Lac-β-CyD was associated with NPC-like HepG2 cells higher than TRITC-β-CyD. In addition, TRITC-Lac-β-CyD was partially localized with endolysosomes after endocytosis. Thus, Lac-β-CyD entered NPC-like HepG2 cells via asialoglycoprotein receptor (ASGPR)-mediated endocytosis and decreased the accumulation of intracellular cholesterol in NPC-like HepG2 cells. These results suggest that Lac-β-CyD may have the potential as a drug for the treatment of hepatosplenomegaly in NPC disease.

## Introduction

The Niemann–Pick type C disease (NPC) is one of inherited lysosomal storage disorders, emerges the accumulation of unesterified cholesterol in endolysosomes. NPC was caused by mutations in either the NPC1 or the NPC2 gene, and usually develops severe neurodegeneration, hepatosplenomegaly and failure to thrive childhood [1–3]. The NPC1 protein is localized in endolysosomes and plays an important role in cholesterol trafficking in cells [3–4]. An excessive amount of unesterified cholesterol accumulation in endolysosomes and a shortage of esterified cholesterol in other cellular compartments are observed. Therefore, the cholesterol sequestration is found to be a crucial factor in developing NPC disease.

Cyclodextrins (CyDs) are non-reducing cyclic glucose oligosaccharides obtained by enzymatic means from starch-containing raw materials and have been used for the enhancement of drugs solubility, stability and bioavailability, etc., through complex formation [5–6]. Recently, 2-hydroxypropyl-β-cyclodextrin (HP-β-CyD) has attracted considerable attention for the treatment of NPC disease [4,7–9]. The administration of HP-β-CyD to *NPC1*-knock out (*Npc1^−/−^*) mice has been reported to remarkably prolong the life span of the mice through a reduction in cholesterol levels [7–9]. Most recently, it was reported that HP-γ-CyD, which has great advantages in biocompatibility compared to HP-β-CyD, can also reduce the cholesterol accumulation in NPC-like cells [10]. However, a high dose of HP-β-CyD or HP-γ-CyD was necessary to exert the pharmacological effects in vivo, since neither HP-β-CyD nor HP-γ-CyD enters cells effectively due to their hydrophilicity and high molecular weight.

Receptor-mediated endocytosis is an attractive approach to enhance the cellular uptake of drugs in target cells. It enables not only high drug concentrations within the cells but also minimum concentration at non-target cells, thereby amalgamating high treatment efficacy with low side effects. Of various receptors, asialoglycoprotein receptors (ASGPR) expressing in abundance on hepatocytes could provide advantages for hepatocyte-selective delivery [11]. Actually, the galactosylation of polymers or lipids has been utilized to develop drug carriers with hepatocyte specificity through ASGPR [11]. Therefore, ASGPR-mediated endocytosis seems to be a promising approach to deliver CyDs to hepatocytes for the treatment of hepatosplenomegaly in NPC disease. Therefore, in the present study, we newly fabricated mono-lactose-appended β-CyD (Lac-β-CyD) and evaluated its cholesterol lowering effect in NPC-like HepG2 cells, cholesterol accumulated HepG2 cells induced by U18666A, as a model of NPC hepatocytes.

## Results and Discussion

### Preparation of Lac-β-CyD

**Figure 1:**
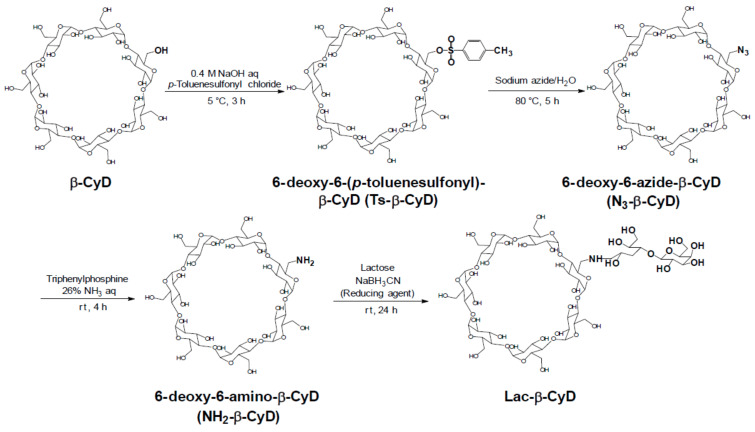
Preparation scheme of Lac-β-CyD.

**Figure 2:**
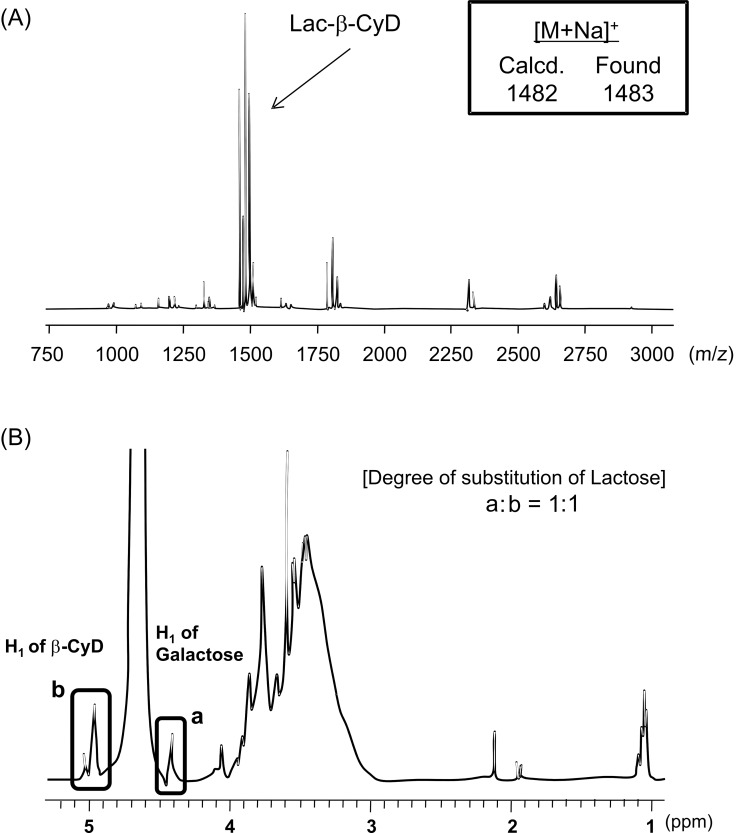
MALDI–TOF MS (A) and ^1^H NMR (B) spectra of Lac-β-CyD.

In the present study, we attempted to prepare Lac-β-CyD as a hepatocyte-selective cholesterol decreasing agent. Figure 1 shows the preparation pathway of Lac-β-CyD. Firstly, we synthesized NH_2_-β-CyD through tosylation, and azidation of primary hydroxy groups of β-CyD, as reported previously [12–13]. Secondly, lactose was modified to NH_2_-β-CyD using the reducing agent cyanotrihydroborate in dimethyl sulfoxide (DMSO) at room temperature for 24 h. No unreacted free lactose was confirmed by thin-layer chromatography (TLC). The MALDI–TOF MS spectrum of Lac-β-CyD showed a peak at *m*/*z* 1483 derived from lactose mono-substituted β-CyD (Figure 2A). There are undesired signals with high molecular weight. Therefore, further elaborate studies are necessary. From the integral values of the anomeric protons of lactose and anomeric protons of glucose in β-CyD obtained by ^1^H NMR analysis (Figure 2B), the degree of substitution of lactose (DSL) was calculated as 1. These results suggest that Lac-β-CyD (DSL1) was almost successfully fabricated.

It is well known that β-CyDs destroy caveolae structures and concomitantly affected various cellular functions through cholesterol depletion [14–15]. In NPC treatment, the interaction of Lac-β-CyD with cholesterol is thought to play a critical role. However, chemical modification of CyDs may often change the inclusion ability to guest molecules. Therefore, to examine whether Lac-β-CyD has inclusion ability or not, we preliminary determined the dissociation constant of Lac-β-CyD with adamantane, which is often used as a guest molecule for β-CyD, using the quartz crystal microbalance method (QCM) in phosphate-buffered saline (PBS, pH 7.4) at 37 °C. As a result, the dissociation constant of Lac-β-CyD with adamantane was 9.8 × 10^−7^ M due to the potent complex formation. Therefore, this preliminary result suggests that the inclusion ability of Lac-β-CyD is still maintained.

### Cytotoxicity of Lac-β-CyD

**Figure 3:**
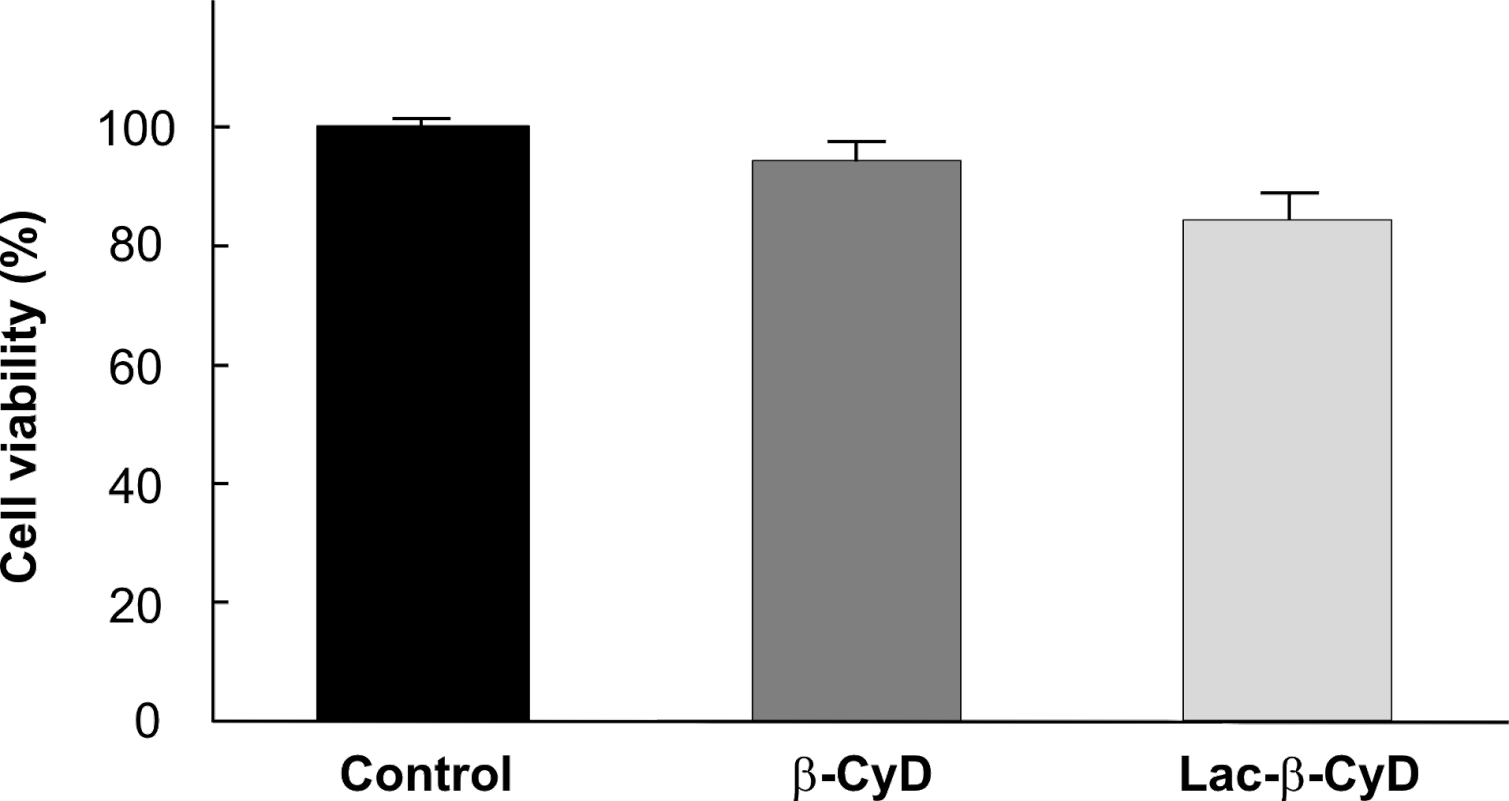
Cytotoxic activity of β-CyDs in U18666A-treated HepG2 cells after treatment for 24 h. U18666A-treated HepG2 cells were incubated with 100 μL of medium containing 1 mM β-CyDs for 24 h at 37 °C. After washing once with PBS, 100 μL of fresh HBSS and 10 μL of WST-1 reagent were added to plates, and incubated for 30 min at 37 °C. Each value represents the mean ± S.E.M. of 6–8 experiments.

To reveal the cytotoxicity of Lac-β-CyD, we examined the WST-1 method (Figure 3). Here, we used U18666A-treated HepG2 cells as NPC-like cells, because U18666A inhibits an intracellular cholesterol trafficking and has the potential to induce NPC disease phenotype [16]. No significant cytotoxicity of Lac-β-CyD was observed in U18666A-treated HepG2 cells at 1 mM for 24 h. The following studies were performed under the experimental conditions.

### Cellular uptake of TRITC-Lac-β-CyD

**Figure 4:**
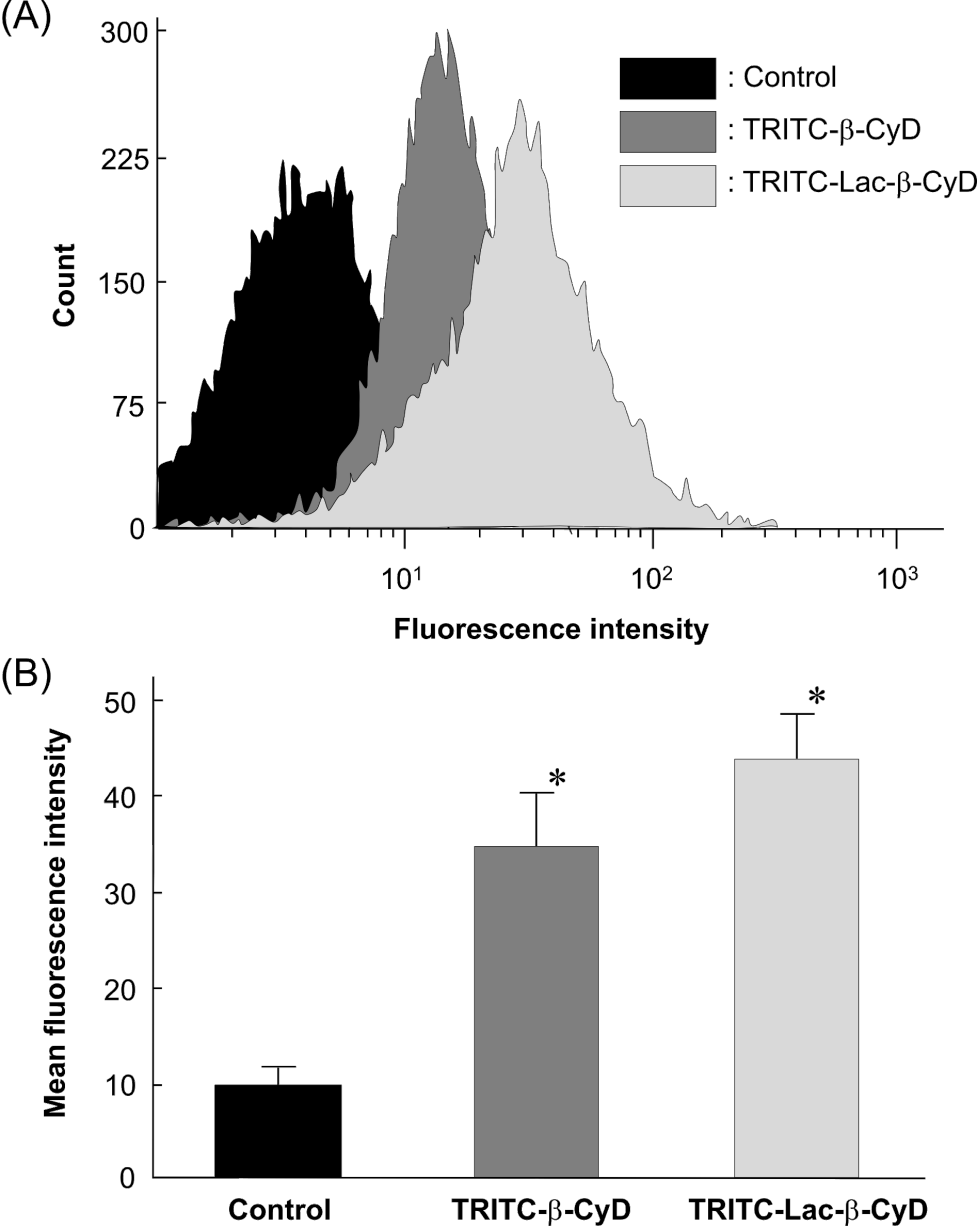
Cellular association of TRITC-Lac-β-CyD in U18666A-treated HepG2 cells after treatment for 24 h. The fluorescence intensity of TRITC in cells was determined 24 h after incubation at 37 °C by a flow cytometer. (A) The experiments were performed independently three times, and representative data are shown. (B) The fluorescent intensity was quantified by CellQuest software. Each value represents the mean ± S.E.M. of 6 experiments. ^*^p < 0.05, compared with control.

To reveal whether Lac-β-CyD can associate with ASGPR-expressing cells, we examined the cellular uptake of tetramethylrhodamine isothiocyanate (TRITC)-labeled Lac-β-CyD after treatment for 24 h in U18666A-treated HepG2 and NPC-like ASGPR-expressing cells, by a flow cytometric analysis (Figure 4). As a result, TRITC-Lac-β-CyD was found to be associated with U18666A-treated HepG2 cells, higher than TRITC-β-CyD, which is lacking the lactose moiety (Figure 4A,B).

**Figure 5:**
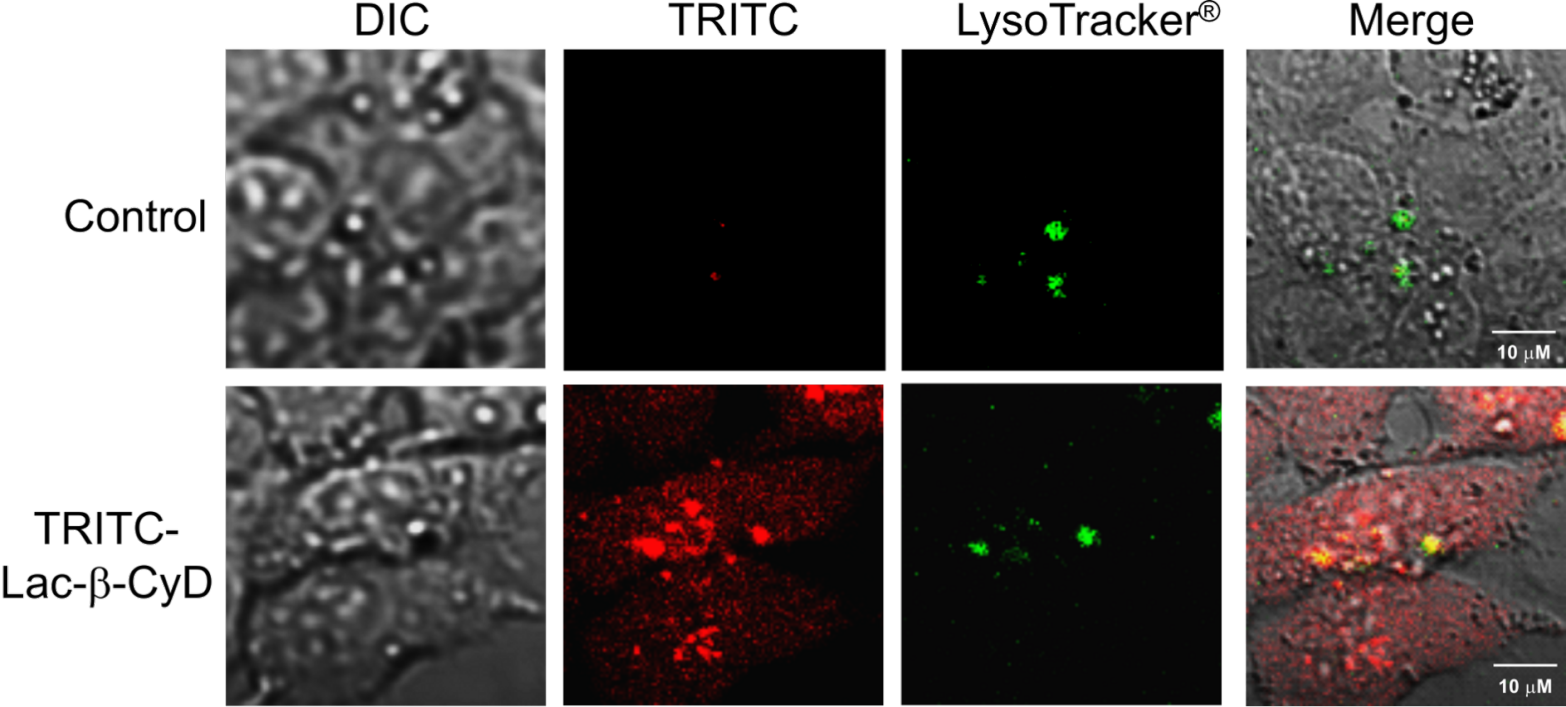
Intracellular distribution of TRITC-Lac-β-CyD in U18666A-treated HepG2 cells. U18666A-treated HepG2 cells were incubated in medium (FBS (−)) with or without 100 μM TRITC-Lac-β-CyD for 24 h. The experiments were performed independently three times, and representative images are shown.

Next, we examined the intracellular distribution of TRITC-Lac-β-CyD by confocal laser scanning microscopy (Figure 5). It should be noted that cellular uptake of TRITC-Lac-β-CyD in U18666A-treated HepG2 cells was observed at 24 h after incubation. In addition, TRITC-Lac-β-CyD was co-localized with endolysosomes stained by LysoTracker^®^. Taken together, these results suggest that Lac-β-CyD localized in endolysosomes of U18666A-treated HepG2 cells.

### Effects of Lac-β-CyD on intracellular cholesterol levels

**Figure 6:**
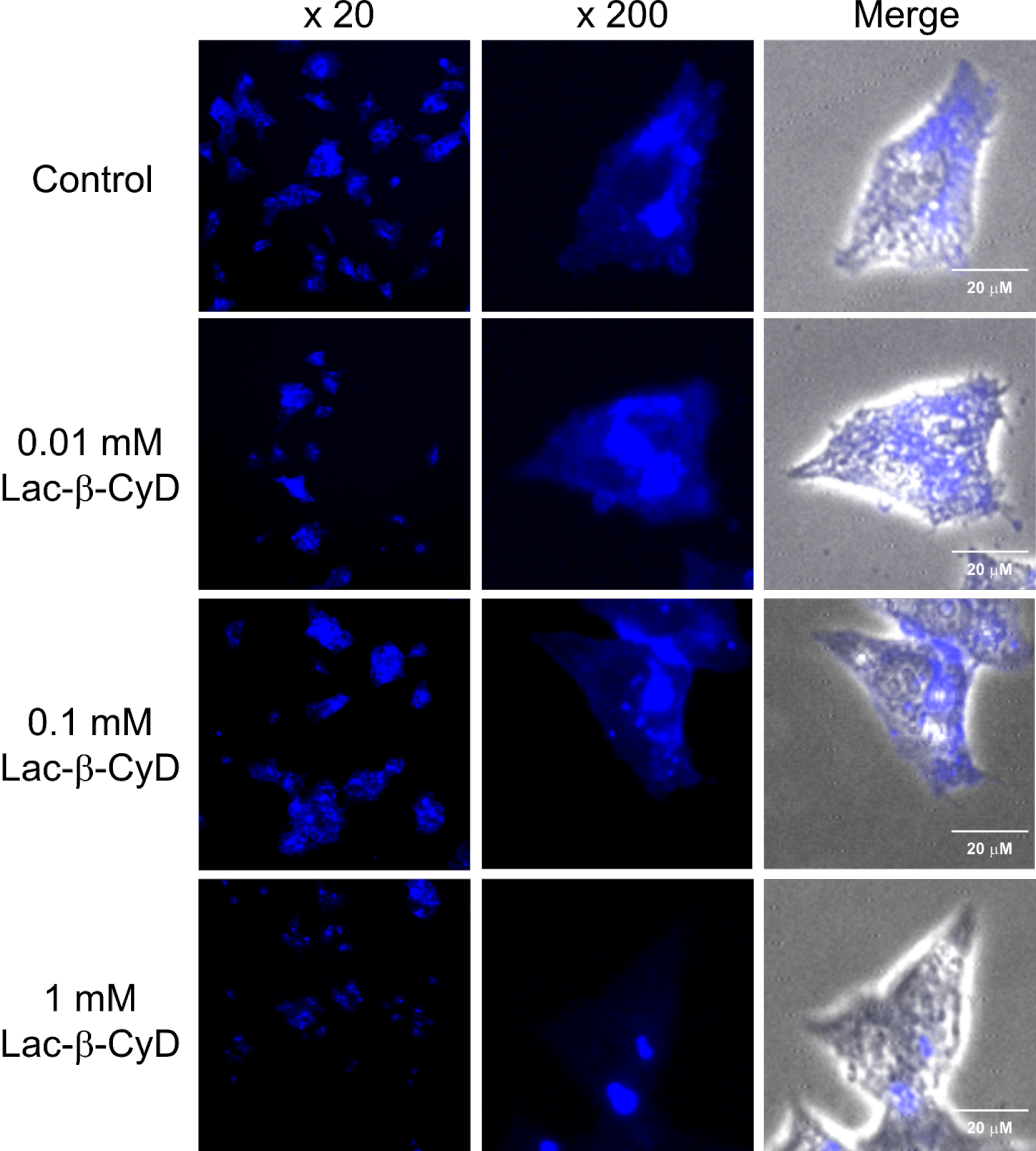
Effect of concentration of Lac-β-CyD on intracellular level of free form of cholesterol in U18666A-treated HepG2 cells. U18666A-treated HepG2 cells were incubated in medium (FBS (−)) with or without Lac-β-CyD for 24 h. The experiments were performed independently three times, and representative images are shown.

We examined the effects of Lac-β-CyD on cholesterol levels in U18666A-treated HepG2 cells using Filipin III, which can bind to unesterified cholesterol specifically. After treatment with 0.01 mM, 0.1 mM and 1 mM Lac-β-CyD for 24 h, the fluorescence intensity of Filipin III was detected by a fluorescence microscope (Figure 6). Herein, the experimental conditions of the treatment with 1 mM β-CyDs for 24 h are reported to decrease cholesterol in endolysosomes in NPC model cells [17–18]. As shown in Figure 6, the treatment with Lac-β-CyD for 24 h decreased the fluorescence intensity derived from Filipin III in a concentration-dependent manner. Taken together, these results suggest that Lac-β-CyD reduced the cholesterol levels in U18666A-treated HepG2 cells through ASGPR-mediated endocytosis.

The proposed mechanism for the cholesterol lowering effects of CyDs in NPC cells are as follows; 1) CyDs may extract the free form of cholesterol from plasma membranes of lipid rafts, and then cholesterol in endolysosomes are transported to plasma membrane to supply, 2) the endocytosed CyDs may transport cholesterol from endolysosomes, and 3) the endocytosed CyDs may elicit a perturbation in endolysosomes membranes through the interaction with membrane components, which would result in the efflux of cholesterol from endolysosomes. In our preliminary study, 1 mM Lac-β-CyD could not extract cholesterol from the plasma membrane of U18666A-treated HepG2 cells within 24 h (data not shown). Therefore, the lowering effects of Lac-β-CyD can be hardly explained by the cholesterol extraction ability from plasma membranes. Recently, Rosenbaum et al. revealed that CyD accelerated the cholesterol trafficking through modulation of the endocytic system, leading to amelioration of cholesterol accumulation in NPC-like cells [19–20]. Although HP-β-CyD and HP-γ-CyD are known to insufficiently enter the cells, Lac-β-CyD endocytosed through ASGPR may have the lowering effects on cholesterol accumulation in U18666A-treated HepG2 cells. However, the role of endocytosed Lac-β-CyD for the cholesterol-lowering effect still remains unclear. To reveal the detailed mechanism of cholesterol lowering effects of Lac-β-CyD, further elaborate studies on not only cholesterol trafficking but also interaction with endolysosomes membranes are necessary.

Autophagy, a bulk digestion system of cytoplasmic aggregated proteins and organelles, plays an important role in regulating NPC disease [21–24]. In NPC disease, accumulation of autophagosomes has elicited even in the basal conditions [25–26]. Therefore, amelioration of impaired autophagy is necessary for the treatment of the NPC disease. Actually, Song et al. demonstrated that HP-β-CyD activated the transcription factor EB, a master regulator of lysosomal function and autophagy, and ameliorated the autophagic function in cells [27]. In addition, Tamura et al. reported that HP-β-CyD elevated LC3-II and p62 levels in NPC1 fibroblasts, indicating the improvement of impaired autophagy by HP-β-CyD [28]. Therefore, it is necessary to investigate whether Lac-β-CyD can improve an autophagy function in NPC-like cells.

Galactose density is one of the critical parameters of affinity to ASGPR. The binding affinity of galactose with ASGPR enhanced 100–1000 fold from mono- to triantennary galactose structure due to cluster effects [29]. Stokmaier et al. reported that the dissociation constant of monosaccharide with ASGPR was 10^−4^ M, while those of triantennary and tetraantennary with ASGPR were 5 × 10^−9^ M and 9 × 10^−9^ M, respectively [29]. Therefore, to provide the more potent recognition ability of Lac-β-CyD to ASGPR, we are preparing the multi-lactose-appended β-CyDs.

## Conclusion

In the present study, we newly fabricated Lac-β-CyD and evaluated its cholesterol lowering effects in NPC-like HepG2 cells. As a result, Lac-β-CyD was endocytosed via ASGPR and decreased the accumulation of intracellular cholesterol in NPC-like HepG2 cells. This result suggests that Lac-β-CyD may have the potential as drug for the treatment of hepatosplenomegaly in NPC disease.

## Experimental

### Materials

β-CyD was kindly gifted by Nihon Shokuhin Kako (Tokyo, Japan). Lactose monohydrate was purchased from Wako Pure Chemical Industries (Osaka, Japan). LysoTracker^®^ Yellow (LysoTracker^®^) was obtained from Life Technologies Japan (Tokyo, Japan). Dulbecco's modified Eagle's medium (DMEM) and fetal bovine serum (FBS) were purchased from Nissui Pharmaceuticals (Tokyo, Japan) and Nichirei (Tokyo, Japan), respectively. Tetramethylrhodamine isothiocyanate (TRITC) was obtained from Funakoshi (Tokyo, Japan). The cell counting kit was purchased from Wako Pure Chemical Industries (Osaka, Japan). The cholesterol cell-based detection assay kit was purchased from Cayman Chemical Company (Ann Arbor MI). All other chemicals and solvents were of analytical reagent grade, and deionized double-distilled water was used throughout the study.

### Apparatus

Nuclear magnetic resonance (NMR) spectra were taken on a JEOL JNM-R 500 instrument (Tokyo, Japan), operating at 500 MHz for protons at 25 °C. The concentration of the sample was 1.5 mg/750 μL in deuterated oxide (D_2_O), and the chemical shifts were given as parts per million (ppm) downfield from that of tetramethylsilane (TMS). MALDI-TOF mass spectra (MALDI–TOF MS) were measured in a positive mode at 25 °C by a JEOL JMS-DX 303 mass spectrometer (Tokyo, Japan).

### Synthesis of Lac-β-CyD

We prepared mono-NH_2_-β-CyD as reported previously [12–13]. Lactose residues were attached to the primary amino group of mono-NH_2_-β-CyD, i.e., mono-NH_2_-β-CyD (1 g) and lactose monohydrate (15.1 g) and sodium cyanotrihydroborate (27.7 g) were dissolved in 3.4 L of 0.2 M borate buffer (pH 7.5) and mixed at room temperature for 24 h. After dialysis using a dialysis membrane, Spectra/pore (MWCO = 1,000), in water at room temperature for 72 h, the sample was concentrated with a rotary evaporator (EYELA N-1000S, Tokyo Rikakikai, Tokyo, Japan), and lyophilized to obtain Lac-β-CyD. The reaction was monitored by TLC (silica gel F_254_, Merck, Whitehouse Station, NJ). Eluent: methanol/water 9:1 (v/v), indicator: *p*-anisaldehyde for sugar and ninhydrin for amino groups. The Lac-β-CyD gave ^1^H NMR spectra consisting of protons of both β-CyD and lactose. The ratios of peak areas of the anomeric proton of β-CyD and protons of the lactose were approximately 1.0, indicating that β-CyD covalently bound to the lactose in a molar ratio of 1:1, as shown in Figure 2B. ^1^H NMR (500 MHz, D_2_O) δ (from TMS), 5.00 (H1, β-CyD), 4.56−4.41 (anomeric proton of lactose), 3.84−3.62 (H3, H5, H6, β-CyD), 3.54−3.43 (H2, H4, β-CyD). The yield of Lac-β-CyD was 2.1%.

### Synthesis of TRITC-Lac-β-CyD

TRITC was attached to the primary hydroxy group of Lac-β-CyD, i.e., Lac-β-CyD (10 mg) and TRITC (1 mg) were dissolved in 400 μL of DMSO and mixed at room temperature for 24 h under the protection from light. Then, the sample was gradually dropped into 50 mL of acetone. After centrifugation (10,000 rpm, 10 min), the precipitant was collected and dissolved with water. The sample was lyophilized to obtain TRITC-Lac-β-CyD.

### Cell culture

HepG2 cells, a human hepatocellular carcinoma cell line, were obtained from Riken Bioresource Center (Tsukuba, Japan). HepG2 cells were grown in DMEM, containing 1 × 10^5^ mU/mL of penicillin, 0.1 mg/mL of streptomycin supplemented with 7.5%, 10% and 10% FBS, respectively, at 37 °C in a humidified 5% CO_2_ and 95% air atmosphere. NPC-like HepG2 cells, which accumulate the cholesterol and sphingolipids in cells, were prepared by the treatment with DMEM containing 1.25 μM U18666A for 48 h.

### Cytotoxicity

In a similar manner as described in [30], cytotoxicity was assayed by the WST-1 method. Briefly, U18666A-treated HepG2 cells were seeded at 3 × 10^4^ cells onto 96-well microplate (Iwaki, Tokyo, Japan), and incubated for 6 h in a humidified atmosphere of 5% CO_2_ and 95% air at 37 °C. Cells were washed once with phosphate-buffered saline (PBS, pH 7.4), and then incubated for 24 h with 150 μL of DMEM containing Lac-β-CyD (0.01, 0.1 or 1 mM) or Tween 20 in a humidified atmosphere of 5% CO_2_ and 95% air at 37 °C. After washing twice with PBS to remove Lac-β-CyD, 100 μL of fresh Hanks’ balanced salt solution (HBSS, pH 7.4) and 10 μL of WST-1 reagent were added to the plates and incubated for 30 min at 37 °C. The absorbance at 450 nm against a reference wavelength of 630 nm was measured with a microplate reader (Bio-Rad Model 550, Tokyo, Japan).

### Cellular association of Lac-β-CyD

Cellular association of Lac-β-CyD was determined by a flow cytometry. After incubation with TRITC-Lac-β-CyD for 1 h in U18666A-treated HepG2 cells, the cells were washed with PBS (pH 7.4) twice and immediately scraped with 1 mL of PBS (pH 7.4). The cells were collected and filtered through nylon mesh. Data were collected for 1 × 10^4^ cells on a FACSCalibur flow cytometer using CellQuest software (Becton-Dickinson, Mountain View, CA).

### Intracellular distribution of Lac-β-CyD

U18666A-treated HepG2 cells (5 × 10^4^ cells/35 mm glass bottom dish) were incubated with 150 μL of DMEM containing 100 μM TRITC-β-CyDs for 24 h. After washing with PBS, 150 μL of LysoTracker^®^ (final concentration: 100 nM) was added and further incubated for 1 h. After the cells were washed, the fluorescence derived from TRITC and LysoTracker^®^ in U18666A-treated HepG2 cells was detected by confocal laser scanning microscopy. The fluorescence intensities were determined by a BZ-II analyzer (Keyence, Osaka, Japan).

### Intracellular distribution of cholesterol

U18666A-treated HepG2 cells (5 × 10^4^ cells/35 mm glass bottom dish) were incubated with 150 μL of DMEM containing Lac-β-CyD (0.01, 0.1, or 1 mM) for 24 h. After washing with PBS, intracellular cholesterol was detected by a cholesterol cell-based detection assay kit (Cayman Chemical Company, Ann Arbor, MI). Briefly, after fixation of the cell by treatment with 150 μL of cell-based assay fixative solution (4% formaldehyde), 150 μL of cholesterol detection assay buffer containing 50 μg/mL of Filipin III was added and further incubated at 37 °C for 1 h. After the cells were washed, the fluorescence derived from Filipin III in U18666A-treated HepG2 cells was detected by a KEYENCE Biozero BZ-8000, a fluorescence microscope. The fluorescence intensities were determined by a BZ-II analyzer (Keyence, Osaka, Japan).

### Data analysis

The experimental results are shown as means ± S.E.M. Significance levels for comparisons between samples were determined with Scheffe's test. The level of statistical significance was set at *p* < 0.05.
